# Current Thoughts of Notch’s Role in Myoblast Regulation and Muscle-Associated Disease

**DOI:** 10.3390/ijerph182312558

**Published:** 2021-11-29

**Authors:** Jeffrey C. Gerrard, Jamison P. Hay, Ryan N. Adams, James C. Williams, Joshua R. Huot, Kaitlin M. Weathers, Joseph S. Marino, Susan T. Arthur

**Affiliations:** 1 Department of Applied Physiology, Health and Clinical Sciences, University of North Carolina-Charlotte, Charlotte, NC 28223, USA; jgerrar1@uncc.edu (J.C.G.); jhay4@uncc.edu (J.P.H.); radams57@uncc.edu (R.N.A.); jwill635@uncc.edu (J.C.W.III); kweath14@uncc.edu (K.M.W.); jmarin10@uncc.edu (J.S.M.); 2 Department of Surgery, Indiana University School of Medicine, Indianapolis, IN 46202, USA; jrhuot@iu.edu

**Keywords:** Hes1, satellite cells, cachexia, Pax7, muscle repair, Wnt, mTOR

## Abstract

The evolutionarily conserved signaling pathway Notch is unequivocally essential for embryogenesis. Notch’s contribution to the muscle repair process in adult tissue is complex and obscure but necessary. Notch integrates with other signals in a functional antagonist manner to direct myoblast activity and ultimately complete muscle repair. There is profound recent evidence describing plausible mechanisms of Notch in muscle repair. However, the story is not definitive as evidence is slowly emerging that negates Notch’s importance in myoblast proliferation. The purpose of this review article is to examine the prominent evidence and associated mechanisms of Notch’s contribution to the myogenic repair phases. In addition, we discuss the emerging roles of Notch in diseases associated with muscle atrophy. Understanding the mechanisms of Notch’s orchestration is useful for developing therapeutic targets for disease.

## 1. Introduction

The evolutionarily conserved signaling pathway Notch is unequivocally essential for cell fate determination during embryogenesis. In adult tissue, Notch is important for regeneration. Activation of this peculiar pathway entails the binding of Notch receptors (Notch 1–4 in mammals) with their ligands (Delta-like 1, Delta-like 4, Jagged1, and Jagged2 in mammals), which causes a series of enzymatic cleavages of Notch receptor. The cleaved and now active intracellular region of Notch (NICD) translocates to the nucleus where it binds to transcriptional regulators CBF-1, Suppressor of Hairless, and Lag-1 CSL (also known as RBP-J). The CSL members release their inhibition which allows for activation of transcription factors, including the basic helix-loop-helix (bHLH) family of transcription factors hairy enhancer of split 1 (Hes/Hey gene family) (in mammals) [1,2]. Notch’s role in skeletal muscle repair is broad yet disagreed upon. The repair of injured skeletal muscle requires the activation of adult skeletal muscle stem cells, termed satellite cells, to sequentially express key bHLH myogenic regulatory factors (MRFs), including myogenic factor 5 (Myf5), myoblast determination protein 1 (MyoD), myogenin, and myosin heavy chain (MHC), which direct myoblast proliferation, differentiation, and ultimately fusion [3]. Notch’s contribution to the muscle repair process is complex and obscure but necessary. It is known that successful myogenesis includes tight regulation of Notch. Understanding the mechanisms of Notch’s orchestration is useful for developing therapeutic targets for disease. There is a conglomeration of skeletal muscle-related diseases associated with defective Notch signaling. The purpose of this review article is to examine the prominent evidence and associated mechanisms of Notch’s contribution to the myogenic repair phases. In addition, we discuss the emerging roles of Notch in diseases associated with muscle atrophy.

## 2. The Public Health Concern of Skeletal Muscle Diseases

Understanding the role of Notch in regulating skeletal muscle repair will provide useful information for the treatment of debilitating muscle-associated diseases. Since skeletal muscle accounts for ~50% of human body mass [4] and is the most metabolic tissue, many chronic diseases, including cardiovascular, pulmonary, metabolic, and cancer, are associated with muscle-related consequences. These deleterious effects will foster a more sedentary lifestyle and result in a poor quality of life, loss of independence, and significant public health consequences. For example, sarcopenia is a condition related to the loss of muscle mass and strength due to age [5]. The reported prevalence of sarcopenia in adults 70.1 years of age was 36.5% [6,7]. It has been suggested that the direct healthcare cost for sarcopenic patients is approximately USD 40.4 billion, showing a significant economic burden [8]. Skeletal muscle is a vital organ, and sarcopenia exerts its negative effects on other systems, such as weakening the cardiovascular system, decreasing bone density, and causing poor glucose regulation, resulting in complete debilitation of the inflicted patient [9]. In addition, our laboratory studied the healthcare costs of the muscle-wasting condition cachexia and found increased hospital stays (6 versus 3 days) and higher costs per stay (USD 4631.30) for patients diagnosed with cachexia [10]. Understanding the pathology of these muscle-associated diseases will better create effective treatment plans. Delineating the skeletal muscle repair process will provide critical information for interpreting pathological findings to devise therapeutic strategies that counteract physical frailty and improve quality of life. This, overall, will reduce the financial burden on the healthcare system that diseases associated with muscle wasting (such as cardiovascular, pulmonary, metabolic, and cancer diseases and acquired immune deficiency) induce.

## 3. Notch during Embryogenesis

Notch’s essentiality was determined during studies of embryogenesis in *Drosophila melanogaster*. Notch signaling is highly expressed in mitotically active cells throughout the various developmental stages. Through cell-to-cell interaction, Notch directs position-dependent development and cell fate decision. This allows for segregating lineages from groupings of precursor cells [11]. For example, Notch is critical in the formation of epidermal cells during the separation of the mesoderm and ectoderm germ layers. In addition, Notch directs the differentiation of embryogenic precursor cells into neuroblasts and dermoblasts within the ectoderm, ultimately deciding the precursor cells’ fate to either neural or epidermal pathways [12].

The process of muscle development during embryogenesis consists of epiblasts being converted to mesoderm, where somites eventually form. The environment around the somites consists of Notch 1 inhibiting MyoD activity. Upon signaling, the Notch-induced myogenic repression is released, and the somites differentiate into dermomyotome, which is the source of muscle precursor cells [13]. However, Notch signaling is critical. If Notch signaling is not present, cell fate decisions cease and there would be no delineation of various tissues, resulting in embryonic death [1].

## 4. Notch and the Adult Skeletal Muscle Tissue

Notch’s action of delineating tissue homeostasis and cell fate decisions is also active in repairing injured adult tissue, including skeletal muscle. Since the mid-1990s, there has been an abundance of published work on the numerous roles of Notch within postnatal skeletal muscle repair [13]. Although Notch is critical for myogenesis in the developing embryo, as well as for adult skeletal muscle repair, the mechanisms for these muscle-related processes may differ. Special attention to the role of the inflammatory process needs to be given when discussing Notch and skeletal muscle repair. Contraction-induced muscle injury (such as exercise) activates a cascade of cellular processes that set the stage for satellite cell activation and initiation of the myogenic pathways. The orchestration of the invading and resident inflammatory cells for satellite cell activation and the muscle repair process is well known [14]; however, although the connection of inflammatory cells to Notch signaling in regulating myogenesis is clear, evidence suggests that there is an antagonistic relationship [15,16,17]. Macrophages recruited to injured skeletal muscle activate satellite cells, possibly by inhibiting Notch signaling [15]. Infiltrating macrophage-derived ADAMST1 (a disintegrin-like and metalloproteinase with thrombospondin type 1 motif) activates satellite cells yet inhibits downstream targets (Hes-1 and Hey-1) by interacting with the NOTCH1 receptor. Although the contextual role of Notch during satellite cell activation and myoblast proliferation is not clear, these data suggest that macrophage activity during skeletal muscle repair inhibits Notch activation and initiation of the muscle repair process [15]. The adversarial relationship between Notch and inflammatory cells was also reported in an ischemic model of muscle injury [16]. Inhibiting Notch signaling through ligand blocking (Delta-like 4) resulted in a 2.5-fold increase in leukocyte recruitment (using CD45^+^ and CD11b^+^ markers) as well as increased presence of leukocyte chemoattractant CXCL1 (chemokine |C-X-C motif|) ligand and recruitment of leukocytes to the ischemic-induced muscle injury site [16]. Furthermore, ingestion of a non-steroidal anti-inflammatory drug (NSAID) following injurious electrically stimulated muscle contractions resulted in greater Notch signaling in activated satellite cells, suggesting that inhibiting the inflammatory process upregulates Notch and affects myogenesis [17]. The contextual relationship of the inflammatory process, Notch signaling, and myogenesis needs to be studied. Investigating cross-talk between inflammatory cells and Notch would provide significant insight on Notch’s role during skeletal muscle repair.

Notch has been identified to be critical in promoting cellular proliferation while inhibiting differentiation, inducing apoptosis, maintaining cellular homeostasis and cellular quiescence, and possibly activating adult skeletal muscle stem cells, (i.e., satellite cells) [2,11,18,19]. The pleiotropic nature of Notch is interesting as Notch has a broad spectrum of influences during postnatal expansion. Through a physical connection of Notch receptors (Notch 1–4) and ligands (Delta-like 1, Delta-like 4, Jagged1, and Jagged2) between two cells, Notch has the ability to influence a cell’s fate and the processes of proliferation, differentiation, and apoptosis [1].

## 5. Notch Promotes Myoblast Proliferation and Inhibits Differentiation

### 5.1. Notch’s Effect on Proliferation and Differentiation Using Cell Culture Models

Notch’s ability to promote proliferation and inhibit differentiation signifies its importance during the early stages of muscle repair. When Notch is inhibited 2 days following muscle injury via the soluble Jagged fusion protein Jagged1-Fc 2, muscle repair is delayed. Interestingly, delayed repair did not occur when Notch was inhibited 5 days post-injury [20]. In addition, there was less early myoblast proliferating myogenic regulator factor Myf5 in 2-day-cultured myogenic cells isolated from single muscle fibers that were treated with Notch inhibitors (gamma secretase inhibitor (GSI) or soluble Jagged 1 fusion protein) [20]. Since muscle repair was affected when Notch signaling was inhibited during the early stages but not during the later stages, this provides evidence of Notch’s importance in the early stages of muscle repair.

There are multiple studies that show increased myoblast proliferation when Notch signaling is force-activated. The study [19] showed, using a cell-tracking dye, that bromodeoxyuridine (BrdU) incorporation was accelerated in myoblasts treated with active Notch 1. In cultured myofiber explants, Notch 1 receptor was present at time 0 (when explants were generated) and co-expression of active Notch and Desmin increased during the first four days of explanation. Increased Notch also coincided with increased Delta 1 and levels of the proliferation marker M-Cadherin. Notch 1 transcripts with the myogenic marker Myf5 were also elevated in proliferating chick embryo limb bud myoblasts [21]. Force activation of Notch increases proliferation, suppresses the myogenic regulatory factor MyoD, and decreases differentiation [22,23,24]. Retroviral overexpression of Hey1 in primary myoblasts reduced MyoD and myotube formation when the myoblasts were differentiated [24]. Accelerated cell cycle replication phases (S, G2, and M phases) were seen in porcine satellite cells treated with the Notch force activator, recombinant human NF kappa-B protein (rhNF-κB). Interestingly, Buas et al. [25] suggested that Notch’s effects may not solely impact proliferation. Using a retrovirus to force-activate NICD in C2C12 cells, Notch was not able to overcome p21-induced G1 arrest, and cell replication stopped but differentiation was still inhibited.

Unique models of Notch inhibition exemplify inhibited proliferation yet accelerated differentiation of myoblasts [22,24,26,27,28,29,30,31,32]. Our laboratory and others have shown promotion of differentiation and impediment of proliferation in the presence of the gamma secretase inhibitors L-685,458 and N-[N-(3,5-difluorophenacetyl)-l-alanyl]-S-phenylglycine t-butyl ester) (DAPT), including a 30% increase in MHC-positive C2C12 myogenic cells and a decrease in the cell cycle progression from G0/G1 to S phase [20,26,31].

### 5.2. Notch’s Effect on Proliferation and Differentiation in Rodent Models

Models of inducible transgenic mice that target Notch signaling have been consistent in their reporting of increased expression of markers of differentiation but inhibited proliferation [18,24,30,33,34,35]. There is increased MyoD and myogenin expression on isolated myofibers and cross-sections of conditional double knockout HeyL/Hes1 mice [24]. Similarly, using the muscle growth model of overload to study myogenic properties, HeyL-KO mice showed decreased proliferation and bias towards differentiation [34]. Mouse models of satellite-cell-specific targeted disruption of Notch 1 and/or Notch 2 generated by crossing Pax7^CreERT2/+^ mice with Notch 1-floxed and/or Notch 2-floxed mice provided significant evidence of Notch 1 and 2′s impact on muscle repair [36]. Seventeen-day proliferating primary myoblasts from the genetically ablated Notch 1 and 2 mice resulted in decreases in the proliferative marker Ki67 but increased myogenin expression, showing accelerated differentiation [36]. In addition, 4 days following cardiotoxin-induced injury, decreases of 16.6%, 20.3%, and 59.0% in the muscle weight of Notch 1-inactived, Notch 2-inactivated, and double knockdown mice, respectively, were found [36]. In addition, there was significant collagen type I expression in all Notch-inactivated mice, with profound fibrosis observed in the double knockdown mice. Notch 1 and Notch 2 coordination is necessary for muscle repair [30].

Inducible depletion of the transcription repressor *Rbpj* (its inhibition is released upon interacting with Notch, and downstream Notch targets are activated) increased myogenin levels by 4.5–7.5-fold and led to an 80% decline in satellite cell Pax7 expression. Moreover, 40 days following Rbpj depletion, the only satellite cells remaining were those with active Notch signaling (where the Rbpj escaped recombination) [33]. Meanwhile, Bjornson et al. [18] demonstrated that Notch inhibition caused satellite cells to skip proliferation and transition directly into differentiation. Following chemical muscle injury induced by barium chloride in *Rbpj*-depleted transgenic mice, the satellite cells activated, did not proliferate, yet terminally differentiated. Bjornson et al. identified that the RBP-Jκ-deficient SCs spontaneously activated, lacked the ability to self-renew, and underwent terminal differentiation. This ultimately resulted in depletion of the satellite cell pool and a poor regenerative capacity, illustrating Notch’s essential role in proliferation [18].

### 5.3. Manipulating O-fucosylation Activity to Study Notch’s Effect on Proliferation and Differentiation

In recent years, methods of manipulating the o-fucosylation activity of Notch receptors have been informative in studying Notch’s role in muscle repair. O-fucosylation controls the interaction of a Notch receptor with its ligands, and hypofucosylation of Notch receptor has been shown to inhibit Notch activity [27,35,37]. Generation of a stable C2C12 cell line with knockdown of O-fucosyltransferase 1 (Pofut1), the protein responsible for o-fucosylation of the Notch receptor, caused decreased expression of the intracellular domain of active Notch (range of 34–87%) and a 66% decrease in downstream *Rbpj* and Hes1 (range between 53% and 71%) but no effect on the full-length Notch receptor [27]. There was a greater decline in Pax7 expression in the Pofut1 knockdown proliferating C2C12 cells yet accelerated differentiation relative to wild-type C2C12 cells. When Pofut1 expression was re-established, cleaved Notch and Pax7 increased 3.4-fold and 5.4-fold, respectively. Moreover, this led to a 72% reduction in myotube formation relative to Pofut1 knockdown C2C12 cells [27].

Similar findings were also seen in a mouse model in which Pofut1 was mutated, resulting in inhibited Notch signaling [35]. After 10 days of differentiating the primary myoblast cultures, the Pofut1-mutated myotubes had a 13% fusion index, and the primary cultures derived from the wild-type mice showed only 9% fusion. In addition, Pofut1 mutation promoted earlier differentiation initiation and upregulated myogenin expression [35].

### 5.4. Manipulating Sialylation Activity to Study Notch’s Effect on Proliferation and Differentiation: Cell Culture and Rodent Models

Recent studies investigated the role of sialylation in the regulation of Notch and myogenesis. Sialylation is active during myoblast proliferation and is decreased during differentiation [38]. Inhibiting sialylation by downregulating α2,6 sialytransferase activity through generation of an st6gal1-knockdown C2C12 cell line results in decreased myoblast proliferation coupled with less Pax7 and a 70% decline in Notch signaling while initiating earlier differentiation [32]. Table 1 displays the literature on models used to study the role of Notch in myoblast proliferation and differentiation.

## 6. Mechanisms of Notch’s Action on Proliferation and Differentiation

### 6.1. Notch’s Effects on Proliferation/Differentiation through Pax7

Cell culture models. The mechanisms that regulate Notch’s activity during myoblast proliferation and differentiation may consist of a collaborative effort to maximize muscle repair. Pax7 and Notch orchestration is a mechanism that regulates myoblast proliferation and differentiation. Pax7 may inhibit differentiation by interacting with MyoD. Active Notch, NICD, was induced in Pax7-expressing satellite cells from the Pax7-CreER/ROSA-N1ICD mouse line. In this overexpressed Notch model, there was a 3–4-fold decrease in MyoD and myogenin [36]. The data suggest that Notch’s activation of Pax7 caused Pax7 to inhibit differentiation by either activating DNA binding and differentiation (ID) proteins (Id2 and Id3) to MyoD or activating proteasome-dependent MyoD [39,44,45]. Silenced PAX7 expression with siRNA in rhabdomyosarcoma cell line RH30 PAX7^+^ cells led to decreased proliferation of the factors ID1, -2, -3, and -4 and sine oculis-related homeobox (SIX1 and -4) but increased MyoD and MyoG [39]. How Pax7 directs ID’s inhibition of MyoD is not clear, but there is evidence that IDs bind to the MRFs’ E-proteins and prevent transcriptional activity [46].

Rodent models. Active Notch, NCID, was induced in Pax7-expressing satellite cells from the Pax7-CreER/ROSA-N1ICD mouse line. In this overexpressed Notch model, there was a 3–4 fold decrease in MyoD and myogenin [36]. The muscular dystrophy with myositis (mdm) mouse model showed a physical interaction of IDs with Ankyrin repeat domain protein 2 (ANKRD2), resulting in inhibited myogenic differentiation [47]. This may be one mechanism of Pax7′s ability to interact with ID to inhibit differentiation.

Notch and Pax7 may regulate myoblast proliferation through the Yes-Associated Protein (YAP), which is known for promoting proliferation and inhibiting differentiation [40]. Overexpressing YAP in decamethonium-induced atrophied chick embryos rescued Notch and Pax7 yet decreased MyoD/myogenin, ultimately resulting in less differentiation [40]. Since ANKRD is a direct target gene of YAP, there may be a relationship between YAP, Pax7, Notch, IDs, and ANKRDs. There are opposing views of Pax7′s mechanism in inhibiting differentiation. It is possible that Pax7 does not inhibit MyoD but rather delays myogenin activity [48]. Nevertheless, the relationship between Notch and Pax7 is critical since Pax7 expression allows for proliferation and prevents differentiation. It should be noted that although Pax7 may inhibit differentiation during postnatal myogenesis, during embryogenesis, Pax7 upregulates MyoD to promote myogenic determination; therefore, the relationship between Pax7 and MyoD should be considered contextual [44].

### 6.2. Notch’s Effects on Proliferation/Differentiation through Hes/Hey

Independent of Pax7, Notch may affect MyoD during regulation of myogenic proliferation and differentiation through the Hes/Hey family or by affecting the cell cycle [22,25,41]. Lahmann et al. [41] believe that the balance of proliferation and differentiation is directed by Hes1 expression. Using real-time imaging of muscle stem cells expressing luciferase reporters for HES1 and MyoD, Lahmann et al. [41] determined the expression oscillations of Hes1 and MyoD with mathematical modeling and predicted that Hes1 oscillation inhibits MyoD expression. The prediction was tested by culturing muscle stem cells with Hes1 mutation, and increased intensity and presence of MyoD bioluminescence was observed, demonstrating altered oscillations of MyoD. Furthermore, overexpression of Hes1 plasmid in primary muscle stem cells inhibits MyoD and myogenin expression, possibly by directly binding to MyoD promoter [41]. It was initially thought that Hey1 inhibits differentiation by directly binding to MyoD, possibly by depriving the bHLH E47 to form a heterodimer with MyoD [25,49,50]. Instead, recent evidence shows that HeyL and Hes1 form heterodimers that bind to myogenin promoters and inhibit differentiation [24]. It should be noted that although the HeyL/Hes1 heterodimer is important for myogenin suppression, this is not the complete story of Notch’s role in muscle repair. Notch may inhibit differentiation through CBF1-dependent and -independent mechanisms [50,51].

### 6.3. Notch’s Effects on Proliferation/Differentiation through Myoblast Transmembranes

Manipulating processes within myoblast proliferation creates unique models to study Notch’s role in myoblast proliferation. The type I transmembrane receptor protein Multiple EGF-like domain 10 (MEGF10) is important for myoblast proliferation. The expression patterns of both Megf10 and Notch correspond during C2C12 myoblast proliferation. Primary myoblasts from MEG10 knockout mice show reduced Notch 1 expression along with poor myoblast proliferation [42]. Immunoprecipitation studies show that Megf1 and Notch 1 interact at their intracellular domains and appear to co-localize. Due to the structural similarity of MEGF10 and Notch ligands, their interaction may be required for activation of Notch signaling [42]. More work is needed to study the interaction of MEGF10 and Notch in regulating myoblast proliferation.

### 6.4. Notch’s Effects on Proliferation/Differentiation through NUMB

The inhibition of Notch by NUMB is another mechanism to describe myogenesis. NUMB is asymmetrically expressed in myogenic daughter cells. It is proposed that NUMB-positive cells that co-express the proliferating marker proliferating cell nuclear antigen (PCNA) inhibit Notch and promote myoblast differentiation. Force activation of Notch with a retroviral Notch 1 construct in myoblasts showed decreased MyoD and desmin expression, while forced expression of Numb resulted in increased desmin levels [19]. It is suggested that elevated Numb levels cause the proliferating myoblasts to exit the cell cycle, express myogenic regulatory factors such as desmin, and undergo myogenic differentiation [19]. The mechanism of how Numb inhibits Notch may be by binding to Notch’s activated C-terminal intracellular domain via NUMB’s N-terminal phosphotyrosine binding (PTB) domain and inducing either endocytosis of Notch receptors and ligands or ubiquitin-mediated protein degradation. Ultimately, NUMB prevents translocation of Notch’s intracellular domain to the nucleus for transcriptional activation [19,50]. Recently, Luo et al. [52] provided a novel finding that NUMB stabilized Notch instead of inhibiting it. Using HeLa, HEK-293T, and SH-SY5Y cell lines, the authors demonstrated that NUMB binds to a region on Notch that is different than the endocytosis/ubiquitination region, followed by NUMB’s activation of a deubiquitinating enzyme, BRCA1-associated protein 1 (BAP1), which binds to Notch 1 and prevents its degradation [52]. Although these observations have yet to be tested during myogenesis, the data provide interesting information on the complexity of Notch and NUMB interactions.

### 6.5. Notch’s Effects on Proliferation/Differentiation through Signaling Pathways

During the transition from myoblast proliferation to the differentiation process, Notch may interact with signaling pathways that promote the later stages of myogenesis. Similar to Notch, the evolutionarily conserved signaling pathway Wnt plays critical roles in cell fate determination during embryogenesis and the regeneration of adult skeletal muscle. During postnatal myogenesis, Wnt promotes the later stages of muscle repair.

Wnt. There is an abundance of evidence describing the critical role of Wnt signaling in controlling myogenic regulatory factor expression in embryogenesis as well as canonical Wnt signaling driving myoblast differentiation during postnatal skeletal muscle repair [53]. Wnt signaling interacts with multiple signaling pathways during muscle repair, including transforming growth factor β (TGFβ) and Notch [53]. Our laboratory and others have demonstrated increased Wnt signaling during muscle regeneration [20,54]. There is increased desmin and reduced Myf5 and proliferation activity in cultured single myofibers treated with recombinant Wnt3a, and premature myotube formation and depleted myogenic progenitors were observed in freeze-injured muscle treated with Wnt3a [20]. This suggests that a balance of Wnt signaling is needed for complete muscle repair. A reciprocal interaction between Notch and Wnt during muscle regeneration occurred as premature increases in Wnt signaling were observed when Notch signaling was inhibited during the proliferation phase and impaired Wnt when Notch was force-activated [20]. A negative feedback loop of these signaling pathways has been observed during tongue development. Genetically mutated mice with epithelial Wnt deficiency resulted in reduced Jagged1 Notch ligand and Hes1 in embryonic tongues [55]. Wnt signaling upregulates Notch which then negatively feeds back to regulate Wnt signaling activity to promote embryonic tongue development. The complex relationship between Notch and Wnt is needed for complete muscle repair. There are numerous possible points of interaction between Notch and Wnt, including GSK3Beta, beta-catenin, Jagged1, Hes1, and Numb [3,20,56,57,58,59,60]. Moreover, Protein Kinase C, which regulates cell cycle progression and affects proliferation and differentiation, may play a role in the Notch–Wnt interaction. Fas-associated death domain (FADD) phosphorylation of Protein Kinase C alpha (PKCα) stabilized Notch 1 and resulted in beta-catenin inhibition [61]. Studies are needed to verify the cross-talk points of Notch and Wnt during skeletal muscle regeneration.

mTOR. Since the mechanistic target of rapamycin (mTOR) pathway regulates the later stages of muscle repair, it is plausible that mTOR and Notch interact during muscle regeneration [62,63,64]. Our laboratory has shown that with Notch inhibition in 4-day myotubes, there is increased phosphorylation of mTOR at Ser2448 and Ser2481, and 4EBPI mTOR signaling corresponds with myotube formation. In addition, PTEN (expression protein upstream of mTOR) was also inhibited, suggesting that Notch may act upstream of mTOR to inhibit mTOR signaling and, ultimately, the later stages of muscle repair [31].

Sonic Hedgehog (Shh). Recent evidence demonstrates Sonic Hedgehog (Shh)’s relationship with Notch to promote proliferation [29]. Shh contributes to myogenesis by promoting proliferation and is known to interact with Notch in proliferating chondrocytes and neuroepithelial cells [29,65], so it is plausible to study whether they co-ordinate with myoblast proliferation. Force activation of Notch (rNotch1) in C2C12 cells upregulates the expression of Shh and its components, including Smo and downstream Gli proteins, suggesting that Notch regulates Shh signaling [29]. When Shh was inhibited with the Smo inhibitor cyclopamine, there was decreased myoblast proliferation and less cell cycle transition from G0/G1 to S phase in C2C12 cells exposed to rNotch1.

Transforming growth factor β (TGFβ). The TGFβ superfamily member Activin A is known to promote cellular proliferation and was found to increase Notch 1 as well as Smo, Gli1, Gli2, and Gli3 [29]. In addition, proliferating C2C12 cells treated with Activin A and the Notch inhibitor DAPT resulted in decreased expression of Shh, Smo, Gli1, Gli2, and Gli3. The authors suggested that Activin A induces the proliferative effects onto Shh through Notch signaling [29].

Post-transcriptional modifications. A recent study has identified a novel mechanism of Notch’s influence on proliferation [66]. N6-methyladenosine (m^6^A) modification is a post-transcriptional modification of mRNA needed for appropriate gene expression and consists of either methylating the mRNA with methyltransferase METTL3 or demethylating with demethylases FTO and ALKBH5. Since it was identified that the active writer m^6^A modification METTL3 influences the proliferation and differentiation of satellite cells. [67], Liang et al. [66] tested whether Notch was affected by the mRNA modifications and influenced myoblast proliferation. Skeletal muscle repair was inhibited in METTL3 conditional knockout with decreased proliferation (less 5-Ethynyl-2′-deoxyuridine (EdU) incorporation into replicating DNA), Pax7-positive cells, and myogenin yet increased fibrosis presence [66]. However, METTL3 knock-in mice showed increased proliferation using Ki67 as well as elevated Pax7-positive cells, myogenin, and myosin expression. The authors suggested that the decreased proliferation in the METTL3 knockout mice could be due to decreased m^6^A methylation of Notch, particularly Notch 2 receptor, Jagged1 ligand, and downstream target Rbpj at the translational level, since there was mRNA expression of those components [66].

Prostaglandins. Prostaglandins regulate myogenesis by affecting myoblast proliferation or myotube fusions [68]. Recently, prostaglandin E2 receptor 2 subtype 2 (PGE2/EP2) inhibited differentiation through Notch signaling [28]. The Notch inhibitor DAPT downregulates EP2 signaling, suggesting that EP2 signaling is downstream of Notch. The addition of PGE2 to Hu5/KD3 cells resulted in decreased myotube formation, while an inhibitor of EP2, TG6-10-1, increased the fusion index. COX-2 produces PGE2, which activates EP2 signaling. There was increased myotube formation in the presence of the COX-2 inhibitor indomethacin in Hu5/KD3 cells [28]. Treatment of mouse primary myoblasts with CAY 10,598 for 48 h resulted in 21% and 17% increases in cell count for PGE2 and EP4, respectively [68]. In addition, there was improved muscle repair as noted with increased Pax7 expression as well as increased cross-sectional area in injured mouse muscles that were injected with PGE2 [69]. These data demonstrate that the PGE2/EP2 signal inhibits differentiation of human muscle progenitors, possibly through Notch 3 [28].

bHLH transcription factor Stra13/DEC1/SHARP-2. The bHLH transcription factor Stra13/DEC1/SHARP-2 is expressed in skeletal muscle and has been shown to be important for regulating cellular differentiation [43]. Stra13 knockout mice showed accelerated myoblast proliferation, poor quality myotubes, and fibrosis within the skeletal muscle [43]. While Notch is important for the early stages of muscle repair, Stra13 contributes to the later stages, including myotube formation, so it is interesting to examine whether Stra13 interfaces with Notch signaling. Interestingly, Notch expression is increased in Stra13-/- mice. Furthermore, when treated with the Notch inhibitor Jagged-Fc fusion protein, there was reduced myoblast proliferation in both WT and Stra13-/- mice but a 20–30% increase in myotube formation. Upon injecting the Notch inhibitor at 7 days following injury and analyzing samples at 10 days post-injury, there was an acceleration of muscle repair [36]. The authors suggested that Stra13 may inhibit Notch signaling, specifically by binding directly to the intracellular domain of Notch and preventing the active region of Notch from interacting with CBF1. Stra13′s ability to inhibit Notch may allow for completion of the repair of injured skeletal muscle.

p53 pathway. Another possible mechanism of how Notch directs proliferation could be cross-talk with the p53 pathway. Tumor suppressor Trp53 (p53) is known to contribute to apoptosis and cell senescence. In addition, p53 promotes myoblast proliferation as well as myoblast survival [70]. Evidence of this is seen with abolished p53 transcriptional activity in primary muscle cells isolated from conditional Rbpj knockout mice with Dll1-induced Notch signaling [71]. This suggests that Rbpj must be activated in order for Notch signaling to upregulate p53 transcriptional activity. The mechanism for the p53 activity by Notch may be due to the binding of Notch’s downstream target, Hey, to the promoter of the p53 antagonist, Mdm2, thereby allowing p53 activity and myoblast proliferation [71].

There are other pathways of interaction with Notch to regulate myoblast proliferation and differentiation; for example, there is evidence of the sine oculis-related homeobox SIX family member Six1 interacting with Pax7 and Notch to inhibit myogenin and ultimately differentiation [72] and primary myoblasts from liver kinase B1 knockout mice promoting proliferation while inhibiting differentiation and expressing Notch 1 and Hes1 [73].

## 7. Conflicting Thoughts on Notch’s Role in Proliferation and Differentiation in Skeletal Muscle

Interesting insights have emerged as to Notch’s role during skeletal muscle repair, specifically that Notch’s function may be contextual or not as critical for proliferation as once thought [2]. Although there is robust evidence of Notch’s importance in inhibiting differentiation and convincing evidence of Notch’s role in promoting proliferation, there are reports that Notch does not participate in myoblast proliferation, such as those showing decreased Notch signaling in primary proliferating myoblasts [2]. Furthermore, evidence shows that Notch was elevated in quiescent satellite cells, reduced within 20 h following injury, and did not elevate again until 4–5 days post-injury [2].

The reasons for these findings could be that only certain targets of Notch signaling contribute to satellite cell activation, such as reductions in Hey1, -2, and -L and Hes1 and -5 in activated satellite cells and increases in Hes6, which is not a direct Rbpj target [2,18]. Alternatively, perhaps there is a non-canonical Notch pathway [23]. Rbpj-null satellite cells still show proliferation and do not inhibit MyoD, so Rbpj may not be required to inhibit differentiation [23,33,49]. Similarly, Notch 1, 2, and 3 receptors may have different functions. While Notch 1 receptor inhibition results in increased differentiation and poor self-renewal, inhibition of Notch 3 receptor resulted in accelerated proliferation and increased self-renewal of satellite cells [74]. Notch 3 may have a specific target gene different than Notch 1 and Notch 2 receptors. Perhaps Notch’s role is a moderator instead of an activator.

## 8. Dysfunctional Notch Signaling

Muscle atrophy occurs within many different pathologies of diseases. These include sarcopenia, sepsis, AIDS, kidney and heart failure, and cancer [75,76]. This occurs through an increase in protein degradation and a reduced capacity for protein synthesis [76]. Older individuals that presented with lower muscle mass and higher amounts of sedentary time had higher all-cause mortality than those that did not [77]. In-patient stays in the ICU resulted in muscle atrophy after 7 days [78]. Around 75% of those patients had muscle atrophy up to six months later [78]. At day 7 and 6 months post-discharge, these patients presented with a decrease in total satellite cell content [78]. Satellite cell activation is integral for muscle hypertrophy [79]. One such pathway is the Notch signaling pathway. Notch signaling preserves the quiescence of satellite cells during homeostatic conditions and maintenance during proliferation [2]. However, Notch signaling can become dysfunctional in many diseases. Understanding the emerging roles of Notch signaling in different diseased states is imperative to developing new therapeutic strategies. Here, we will discuss the implications of Notch signaling within the musculoskeletal environment in aging, cachexia, muscular dystrophy, and diabetes.

## 9. Aging and Vitamin D deficiency

Aging is accompanied by malnutrition [80,81], satellite cell dysfunction [18,82,83], decreased anabolic hormone secretion [80,84], increased inflammation [18,80,85], and morphological alterations of the neuromuscular junction [80,86]. Collectively, this leads to sarcopenia and dynapenia, characterized by the gradual decline in skeletal muscle mass and strength, respectively [75]. The loss of skeletal muscle mass and strength impedes physical functioning and the ability to perform daily activities, resulting in a loss of personal independence [87]. Importantly, physical activity throughout the lifespan may preserve skeletal muscle mass and strength, thus delaying loss of physical function [88]. However, a better understanding of the cellular and molecular alterations that contribute to sarcopenia may uncover novel targets for therapeutic and pharmacological intervention to preserve muscle mass and function.

Malnutrition encompasses undernutrition, overnutrition, and nutrient deficiencies [89]. One such deficiency that may contribute to sarcopenia is that of vitamin D [80]. Vitamin D is produced by the skin or absorbed by the intestines [80,90]. The skin plays a large role in this deficiency due to skin atrophy associated with aging [80]. In elderly populations, vitamin D deficiencies have been associated with muscle weakness and a higher risk of falling [81]. Vitamin D receptors (VDRs) are inversely related to age [91]. One potential target is the Notch signaling pathway and its interaction with vitamin D. Vitamin D plays an important role in the skeletal muscle environment as it targets skeletal muscle VDRs and modulates the muscle repair process [81]. VDRs are expressed at low levels within uninjured muscle fibers but become expressed in injured muscle fibers and satellite cells [91,92].

### 9.1. Vitamin D Deficiency in Mice and Rats

In C2C12 cells, treatment with vitamin D showed an increase in myotube size through downregulation of myostatin [93]. This response was also paired with an anti-proliferative effect in myoblasts [93]. Overexpression of VDR demonstrated increased recruitment of Pax7+ satellite cells when compared to a control [94]. Vitamin D deficiency in old-age Wistar rats showed a decrease in Notch signaling activation, though its exact mechanisms are not fully understood [81]. Notch mRNA expression did not change, while Notch 1 protein expression was reduced [81]. Domingues-Faria et al. [81] proposed a potential explanation that there is a decrease in proteolytic processing of the Notch receptor that involves a disintegrin and metalloprotease domain (ADAM) proteases or the gamma secretase complex. This relationship was examined in bone marrow precursor cells [95]. The reduction in Notch signaling was confirmed by the downregulation of the downstream target hairy enhancer of split 1 (Hes1).

### 9.2. Vitamin D Deficiency in Humans

In human skeletal muscle, vitamin D3 treatment inhibited myoblast proliferation by expressing cyclin-dependent kinase (CDK) inhibitor proteins p27 and p21 to control cell cycle progression [96]. Myoblast differentiation was downregulated along with a decreased expression of myoblast determination protein 1 (MyoD), myogenin, and Myocyte Enhancer Factor 2C (MEF2C). In addition to these findings, Hes1 was upregulated while the Notch downstream effectors Hes6 and hairy/enhancer-of-split related with YRPW motif protein 1 (Hey1) were downregulated [96]. An explanation for this anti-proliferative effect is that it aids in maintaining the SC pool along with the potential of self-renewal [97]. Myoblasts that have not undergone proliferation or differentiation could then return to a quiescent state [96].

Notch signaling is upregulated by vitamin D and could be promising for maintaining satellite cell populations, which are reduced in diseases such as sarcopenia. The complexities surrounding the differences between effects on humans and effects from in vivo and in vitro experiments on mice and rats should be explored.

## 10. Age-Related Notch Signaling Deficiencies

Inadequate muscle repair may be attributed to the age-related decline in Notch signaling. This is caused in part by a decline in the upregulation of Notch [98]. Carlson et al. [99] showed that in old human myofibers, the Notch signaling ligand Delta was decreased in comparison to younger myofibers. Genes that regulate Notch expression, including Notch 1, Jagged1, and Delta1, present with a decrease in their expression in older human skeletal muscle when compared to younger skeletal muscle [100].

### 10.1. Notch Signaling and FGF-2

A proposed contributor to the reduction in Notch signaling is the downregulation of classical mitogen-activated protein kinase (MAPK) signaling with increasing age [82]. The MAPK/phosphate extracellular signal-regulated kinase (pERK) signaling pathway is necessary for canonical activation of Notch [99]. Insufficient Notch activation in older satellite cells showed a decrease in MAPK activation. MAPK activation in the presence of a gamma secretase inhibitor, through fibroblast growth factor 2 (FGF-2), suggests that MAPK signals upstream of Notch [99]. Satellite cell regenerative responses were blockaded in response to this forced activation of MAPK [99]. Notch signaling and Delta expression are regulated by activation of the fibroblast growth factor 2 (FGF-2)/fibroblast growth factor receptor 1 (FGFR-1) pathway, which subsequently activates MAPK [99,101]. There is an attenuation of FGFR-1 that coincides with increasing age [99]. Activation of FGFR-1 with p38 MAPK inhibition rescues the SC niche [99]. FGF-2 has been shown to be elevated in aged muscle fibers along with MAPK [99,102]. The reason for the elevation in FGF-2 is that it becomes a compensatory signaling stimulus to repair muscle [99,102]. For this reason, elevated levels of FGF-2 have been implicated in modulating SCs’ quiescent state [99,102]. Sprouty 1 (spry1) is an FGF antagonist that, when deleted from aged mice, caused a 50% decrease in SCs compared to control mice [102]. SCs that present with higher levels of spry1 remain in quiescence [102].

### 10.2. Notch and TGF-B

Aged myofibers hamper regeneration upon injury due to a failure to activate Notch signaling and increase its activation of transforming growth factor beta (TGF-B), which hampers muscle regeneration [82]. To have an adequate muscle repair response, Notch signaling must coordinate with the signaling cascade of TGF-B [102]. Notch signaling acts antagonistically on TGF-B by inhibiting its downstream effector phosphorylated Sma- and Mad-related proteins (pSMAD3). pSMAD3 upregulates CDK inhibitors P15, P16, P21, and P27 [103]. These proteins are responsible for inducing arrest of the cell cycle [104]. P16 is a protein of interest because of its activation through the INK4A locus. When activated, its expression inhibits the phosphorylation of retinoblastoma protein (Rb). By inhibiting the phosphorylation of Rb, the protein E2F cannot signal genes that are mandatory for cell cycle progression from the G1 phase to the S phase [104]. It is for this reason that p16 has been implicated in the geroconversion of SCs [105]. Geroconversion puts SCs in a pre-senescent state; then, when forced to break quiescence, they undergo accelerated senescence [105].

In addition to the arrest of the cell cycle, SCs undergo mitotic catastrophe due to decreases in Notch signaling with age [71]. Mitotic catastrophe is cellular death that occurs during mitosis when DNA damage or malformation of spindle complexes was not halted during previous checkpoints in the cell cycle [106]. p53 is responsible for controlling the cell cycle or inducing apoptosis when damage to DNA has occurred [104]. Notch signaling has been implicated in controlling p53 by inhibiting Mdm2 at its promoter gene through its downstream effector Hey1 [71]. p53 has been implicated in antagonizing levels of p16INK4a through different cell types. However, a physiological threshold has not been determined at which p53 suppresses p16INK4a [71].

Notch signaling must be tightly regulated for adequate maintenance of satellite cells. In the absence of Notch signaling, compensatory mechanisms that control the senescence of satellite cells become apparent.

## 11. Anabolic Hormone Decrements

Androgen receptors are expressed in satellite cells within skeletal muscle [107]. Testosterone regulates Notch signaling through the activation of p38 MAPK through testosterone’s regulation of myostatin [108]. Testosterone and estrogen build up the satellite cell pool at the onset of puberty [109]. Mind bomb 1 (Mib1) is responsible for activating the Notch signaling pathway through the activation of androgen and estrogen receptors [109]. Without this activation from testosterone and estrogen, satellite cells do not undergo self-renewal after injury and further deplete reserves [109]. Satellite cell pools were rescued through a sex hormone-Mib1-Notch axis [109].

Testosterone levels steadily decline with aging, resulting in reduced androgenic signaling and implanting low testosterone in sarcopenia [84]. In males, circulating testosterone levels decline at a rate of 1% each year from age 30 [84]. In females, testosterone levels start to decline between the ages of 20 and 45 [84]. The recent literature suggests that there is an age-related decrease in Mib1 [110]. This decrease is suggested to be the cause of the atrophy of type II muscle fibers in an Actn3-dependent manner [110]. Mib1 binds directly to Act3n and begins the process of ubiquitination [110]. Through Notch inhibition, it was concluded that Notch signaling is not necessary for the maintenance of type II skeletal muscle throughout the lifespan [110]. Given that there was an association of Mib1 and Notch signaling, the lack of Notch signaling should not be discounted in its role in age-related maintenance and sarcopenic associations.

## 12. Cachexia

Cachexia is the progressive wasting of muscle mass and adipose tissue associated with chronic diseases such as cancer, chronic obstructive pulmonary disease, chronic heart failure, chronic kidney disease, and AIDS [111,112]. Pro-inflammatory pathways are thought to be a main contributor in cancer cachexia (CAC) [113]. Tumor necrosis factor-alpha (TNF-α), a prognosticator of cachexia, has been shown to regulate Notch signaling within cancer development and metastasis [113]. In mice injected with K7M2 and K12 osteosarcoma lineages, Notch signaling became dysfunctional in the tibialis anterior muscle and the tumors [113]. Notch signaling was inhibited through the use of MK-0752, a gamma secretase inhibitor. Injections were dosed at 50 mg/kg and injected three times per week for 4 weeks. However, upregulated levels of Notch were found within the muscle tissue and were correlated with higher levels of TNF-α [113]. It is thought that there is a spillover effect of TNF-α in the bloodstream from tumorigenesis, which activates Notch signaling within the muscle [113]. This activation of Notch keeps satellite cells in a quiescent state with competing signals to activate myogenesis [113]. In this prolonged quiescent state, muscle tissue can no longer regenerate, leading to atrophy and senescence [113]. Mice that had Notch signaling inhibited showed reduced muscle atrophy when compared to a control. Tumor size was not affected by Notch inhibition [113]. In addition to these findings, K7M2 tumors produced exosomes that could potentially contain miRNA to activate Notch without cell-to-cell contact [113]. Exosomes released by the tumors in the K7M2 mice were isolated and cultured with muscle-derived stem cells. Notch 1 and Hes1 mRNA was found to be subsequently elevated [113]. More research is needed to elucidate a relationship between Notch inhibition and cachexia improvement.

In human sarcoma tumors, it was found that Notch signaling was increased [114]. In tumors of cachectic and non-cachectic sarcomas, those with cachexia exhibited Notch dysfunction [114]. Co-cultured samples agree with Mu et al. [113] that Notch signaling inhibits muscle differentiation due to higher expression of paired box 7 (Pax7+) cells. Contrary to the findings of Mu et al. [113], pro-inflammatory cytokine gene expression was not elevated between groups [114]. Specifically, TNF-α fell below detectable levels [85]. A limitation of this study was the low sample size.

In non-cancer cachectic states such as congestive heart failure or chronic kidney disease, there is an increase in the renin–angiotensin system leading to an increase in angiotensin II [115]. Angiotensin II type 1 receptors (ATR1) are highly expressed in satellite cells [115]. When overexpressed, angiotensin II binds to its receptor and inhibits Notch signaling [115]. By halting the self-renewal capacity, skeletal muscle enters an insurmountable regenerative decline [115]. Satellite cell pools will be depleted and cannot be replenished [115]. Angiotensin II infusions with cardiotoxin (CTX) injections lead to a decrease in CSA and muscle weight in mice [115]. The number of Pax7+ and myoblast determination protein (MyoD)-expressing satellite cells was also reduced [115]. The exact mechanism of how angiotensin II elucidates Notch inhibition in muscle is unknown. Future research should make this a focus.

## 13. Muscular Dystrophy

Muscular dystrophy is a muscle-wasting disease characterized by a genetic deficiency causing a lack of dystrophin, resulting in muscle weakness, contractures, regenerative insufficiency of the muscle, and ultimately death [116,117,118,119]. Notch signaling has been controversially implicated in the disease’s progression [116]. Cytokine inflammation induction of TNF-α and interleukin 1 beta (IL1B) triggers the transcription of nuclear factor kappa-light-chain-enhancer of activated B cells (NF-kB). TNF-a and NF-kB work together to suppress muscle regeneration in muscular dystrophic mice (dmx) [116]. TNF-a and NF-kB also work in tandem to suppress Notch 1 signaling [111]. It has been shown that NF-kB is required for suppression [116]. The mechanisms by which NF-kB accomplishes this remains unclear. TNF-α signaling mediates Notch signaling epigenetically by recruiting Ezh2 for short-term histone methylation within 48 h [117]. Long-term suppression is preserved by Dnmt-3b methylation at the Notch 1 promoter through TNF-a recruitment [116]. This long-term suppression contributes to the poor regeneration of the satellite cell pool [116]. Consistent with the previous findings, blockading Notch signaling in satellite cells causes exacerbation of the muscular dystrophy phenotype in mice [117]. Notch loss of function within satellite cells presented with premature differentiation and reduced proliferation [117].

Notch signaling was elevated in human muscular dystrophy cells due to increased IL-1B [118]. IL-1B has mitogenic effects in human non-muscular-dystrophic muscle cells (HNDMCs) and no effect in human dystrophic muscle cells (HDMCs) [118]. The expression of its gene IL-B and its receptor 1L1R1 showed no difference between both cell types [118]. The differences came from the amount of Jagged1 and Notch expression between HNDMCs and HDMCs. Jagged1 and Notch 3 were upregulated more in HDMCs than in HNDMCs. Notch 1 levels in both groups were low and Notch 4 was undetectable. IL-1B was found to upregulate Jagged1 during periods of inflated cytokine production [118]. This overstimulation of Jagged1 halts IL-1B’s progression of the cell cycle by activating Notch 3 receptors [118]. Further research needs to explore whether findings present with a Notch 1 decrease and, if so, whether there is also an increase in Notch 3 due to Jagged1 activation. It is believed that Notch 3 provides an antagonistic effect on Notch 1, further suppressing the ability of Notch 1 to induce proliferation and activation of satellite cells [118]. Gallot et al. found that myeloid differentiation primary response 88 (MyD88) is necessary to prevent aberrant Notch activation [119]. This is due in part to MyD88 having an inhibitory effect on the IL-1 receptor family [119]. When ablated, Notch signaling is upregulated; however, this coincides with a decrease in satellite cell numbers [119]. Notch signaling between humans and mice is in conflict about the interaction of IL-1B and its effect on Notch. Epigenetic regulation of Notch by Ezh2 could be a starting point to narrow down the difference of mechanisms between humans and mice.

## 14. Diabetes

Diabetes is a disease characterized by the dysregulation of glucose metabolism affecting multiple organ systems [120]. Mice injected with streptozotocin will become diabetic through the destruction of pancreatic B cells [120]. Streptozotocin is a chemotherapy drug for pancreatic cancer but is highly toxic in mice [121]. In mice with induced diabetes, the satellite cell regenerative capacity was impaired due to an upregulation of myostatin and its TGF-B receptors [120]. This upregulation promotes the phosphorylation of Smad3, which in turn activates its downstream target p15, a cell cycle inhibitor [120]. This relationship between TGF-B and Notch signaling was observed previously in aging. Inhibition of TGF-B receptors restored the regenerative capacity of satellite cells, though Notch’s involvement in this process was unexplored. Further studies should look to identify Notch’s role in muscle repair in a TGF-B/pSmad3-dependent manner within the diabetic environment.

### 14.1. Type 1 Diabetes

Decreases in satellite cell number along with regenerative capacity were found in humans and Akita mice with type 1 diabetes [122]. In both humans and Akita mice, Notch signaling was elevated above normal levels [122]. This elevated level keeps satellite cells from activating and continuing their progression towards myogenicity [122]. Muscle repair is then limited to only what is available, which in turn depletes reserve satellite cells [122]. After a bout of downhill running, biomarkers of myogenesis were lower than those in the control group along with a lower satellite cell content [122]. In comparison with humans with and without type 1 diabetes, it was found that those with type 1 diabetes had lower satellite cell numbers [122]. The results show a difference between humans and mice in the activation of Notch. In humans, Delta-like ligand 1 (DLL1) was significantly more elevated than in the mouse counterparts. This leads to speculation that Notch in mice must be triggered another way than Notch–ligand contact [122]. In the murine model, it was observed that Notch is activated by Jagged1 ligands [122]. It was not clear which mechanism was responsible in humans for the increased Notch activation [122]. A proposed theory by D’Souza et al. is that an abundance of matrix metalloproteinase activity signals Notch due to impairments of extracellular matrix remodeling in type 1 diabetic skeletal muscle [122].

### 14.2. Type II Diabetes

Dysfunctional Notch signaling appears to alter insulin signaling in c57bl/6j mice with type II diabetes [123]. Poddar et al. [124] found in a subsequent study that in mice with diabetes, miR-449a levels were decreased. When activated, miR-449a binds to and inhibits Jagged1 expression, leading to a decrease in Notch signaling and its downstream effectors [124]. It was found that Jagged1, NICD, Hes1, and Hey1 levels were significantly decreased with miR449-a activation [124]. Human primary skeletal muscle cells were grown and cross-referenced by immunofluorescence, confirming a decrease in miR449-a activation [124]. By inhibiting Notch signaling in this manner, insulin signaling in the muscle was restored. This was confirmed by the inhibition of Notch using DAPT.

The diabetic muscle environment seems to be one characterized by an overstimulation of Notch signaling [123,124]. Due to the difference in the activation of ligands between humans and mice, research should be focused on the determination of these differences. Inhibition of Notch restored insulin signaling with the muscles [123,124]. In light of this relationship, Notch’s relationship with TGF-B must be taken into account [120]. Inhibition of Notch may restore insulin signaling, but would this inhibition interfere with the balance of TGF-B/pSmad and Notch regarding muscle repair? Future research should take this into consideration.

## 15. Conclusions 

The Notch signaling pathway is a complex cell signaling pathway that is vital to the early formation of life in the embryonic stage and the maintenance of it in the postnatal niche [13]. In the context of postnatal myogenesis, the discourse of its exact roles is controversial. Notch’s role in postnatal myogenesis is that of myoblast proliferation and inhibiting differentiation. Notch must be tightly balanced to regulate muscle regeneration. Activation of Numb may repress Notch in order to initiate myoblast differentiation [19]. Stra13 has been proposed to inhibit Notch to complete muscle repair [43]. If there is an imbalance between TGF-B and Notch, premature senescence can occur due to the induction of CDK-inhibitors—specifically p16 [103,105]. Emerging thoughts have come about that Notch signaling is not as critical for proliferation as previously considered and that its actions are relative to the environment [2]. However, it can be argued that the body of literature disagrees with this notion. Further research should focus on alternative ways that Notch is activated non-canonically. Other avenues could also focus on the exact mechanisms of interplay between Notch and Wnt to coordinate muscle repair [3,18,55,56,57,58,59].

In the state of chronic disease of the muscle, Notch signaling becomes dysfunctional. This is due to a reduction over time with age [98,99], imbalances between Notch and TGF-B signals [103], and decreases in androgens over time [84]. On the contrary, in cases of cachexia [114,115], muscular dystrophy [118], and diabetes [123,124], Notch signaling can become overexpressed. This could lead to a depletion of the satellite cell pool by keeping satellite cells in quiescence under regenerative pressure and inadequately repairing damaged tissues [105]. Due to the intricacies between C2C12, murine, rodent, and human models, more research is needed to effectively come up with therapeutic modalities to improve quality of life.

## Figures and Tables

**Table 1:** Summary of recent evidence on models that investigated Notch’s role in myoblast proliferation and differentiation.

Model	Observation of Myoblasts	References (Cell Culture or Rodent Models)
Force activation of Notch in satellite cells	↑ proliferation ↓ differentiation	Qin (2013) [22] (cell culture), Vasyutina E (2007) [23] (cell culture) Noguchi (2019) [24] (rodent)
NICD (overexpressed Notch) to inhibit Pax7 in Pax7-expressing cells	↓ differentiation	Wen et al. [36] (rodent)
Silenced Pax7 w/siRNA in RH30 PAX7+ cells	↓ proliferation ↑ differentiation	Skrzypek et al. [39] (cell culture)
Overexpressing YAP in atrophied chick embryos	↓ differentiation	Esteves de Lima et al. [40] (rodent)
Hes1 mutation plasmid in primary muscle stem cells	↓ differentiation	Lahmann et al. [41] (cell culture)
MEGF-10 knockout in primary myoblasts	↓ proliferation	Saha et al. [42] (rodent)
Smo inhibitor on Shh in C2C12 cells	↓ proliferation	Ma et al. [29] (cell culture)
Stra13 knockout in mice Active Stra13 inhibits Notch in mice	↑ proliferation ↓ proliferation ↑ differentiation	Sun et al. [43] (rodent)
Gamma secretase inhibitor (DAPT) inhibits PGE2/EP2	↑ differentiation	Sakai-Takemura et al. [28] (cell culture)
Inhibiting sialylation Active sialylation	↓ proliferation ↑ proliferation	Vergé (2020) [32] (cell culture)

## Data Availability

Not applicable.

## References

[1] Hori K., Sen A., Artavanis-Tsakonas S. (2013). Notch signaling at a glance. J. Cell Sci..

[2] Mourikis P., Tajbakhsh S. (2014). Distinct contextual roles for Notch signalling in skeletal muscle stem cells. BMC Dev. Biol..

[3] Tsivitse S. (2010). Notch and Wnt signaling, physiological stimuli and postnatal myogenesis. Int. J. Biol. Sci..

[4] Short K.R., Vitonne J.L., Bigelow M.L., Proctor D.N., Nair K.S. (2004). Age and aerobic exercise training effects on whole body and muscle protein metabolism. Am. J. Physiol. Endocrinol. Metab..

[5] Morley J.E., Baumgartner R.N., Roubenoff R., Mayer J., Nair K.S. (2001). Sarcopenia. J. Lab. Clin. Med..

[6] Brown J.C., Harhay M.O., Harhay M.N. (2016). Sarcopenia and mortality among a population-based sample of community-dwelling older adults. J. Cachexia Sarcopenia Muscle.

[7] Dhillon R.J., Hasni S. (2017). Pathogenesis and Management of Sarcopenia. Clin. Geriatr. Med..

[8] Goates S., Du K., Arensberg M.B., Gaillard T., Guralnik J., Pereira S.L. (2019). Economic Impact of Hospitalizations in US Adults with Sarcopenia. J. Frailty Aging.

[9] Arthur S.T., Cooley I.D. (2012). The effect of physiological stimuli on sarcopenia; impact of Notch and Wnt signaling on impaired aged skeletal muscle repair. Int. J. Biol. Sci..

[10] Arthur S.T., Noone J.M., Van Doren B.A., Roy D., Blanchette C.M. (2014). One-year prevalence, comorbidities and cost of cachexia-related inpatient admissions in the USA. Drugs Context.

[11] Artavanis-Tsakonas S., Rand M.D., Lake R.J. (1999). Notch signaling: Cell fate control and signal integration in development. Science.

[12] Bray S.J. (2006). Notch Signaling: A simple pathway becomes complex. Nat. Rev. Mol. Cell Biol..

[13] Cossu G., Tajbakhsh S., Buckingham M. (1996). How is myogenesis initiated in the embryo?. Trends Genet..

[14] Tidball J.G. (2011). Mechanisms of Muscle Injury, Repair, and Comprehensive Physiology. Compr. Physiol..

[15] Du H., Shih C., Wosczyna M.N., Mueller A.A., Cho J., Aggarwal A., Rando T.A., Feldman B.J. (2017). Macrophage-released ADAMTS1 promotes muscle stem cell activation. Nat. Commun..

[16] Al Haj Zen A., Oikawa A., Bazan-Peregrino M., Meloni M., Emanueli C., Madeddu P. (2010). Inhibition of delta-like-4-mediated signaling impairs reparative angiogenesis after ischemia. Circ. Res..

[17] Mackey A.L., Rasmussen L.K., Kadi F., Schjerling P., Helmark I.C., Ponsot E., Aagaard P., Durigan J.L., Kjaer M. (2016). Activation of satellite cells and the regeneration of human skeletal muscle are expedited by ingestion of nonsteroidal anti-inflammatory medication. FASEB J..

[18] Bjornson C.R., Cheung T.H., Liu L., Tripathi P.V., Steeper K.M., Rando T.A. (2012). Notch signaling is necessary to maintain quiescence in adult muscle stem cells. Stem Cells.

[19] Conboy I.M., Rando T.A. (2002). The regulation of Notch signaling controls satellite cell activation and cell fate determination in postnatal myogenesis. Dev. Cell.

[20] Brack A.S., Conboy I.M., Conboy M.J., Shen J., Rando T.A. (2008). A temporal switch from notch to Wnt signaling in muscle stem cells is necessary for normal adult myogenesis. Cell Stem Cell.

[21] Delfini M.C., Hirsinger E., Pourquié O., Duprez D. (2000). Delta 1-activated notch inhibits muscle differentiation without affecting Myf5 and Pax3 expression in chick limb myogenesis. Development.

[22] Qin L., Xu J., Wu Z., Zhang Z., Li J., Wang C., Long Q. (2013). Notch1-mediated signaling regulates proliferation of porcine satellite cells (PSCs). Cell Signal..

[23] Vasyutina E., Lenhard D.C., Birchmeier C. (2007). Notch function in myogenesis. Cell Cycle.

[24] Noguchi Y.T., Nakamura M., Hino N., Nogami J., Tsuji S., Sato T., Zhang L., Tsujikawa K., Tanaka T., Izawa K. (2019). Cell-autonomous and redundant roles of Hey1 and HeyL in muscle stem cells: HeyL requires Hes1 to bind diverse DNA sites. Development.

[25] Buas M.F., Kadesch T. (2010). Regulation of skeletal myogenesis by Notch. Exp. Cell Res..

[26] Dahlqvist C., Blokzijl A., Chapman G., Falk A., Dannaeus K., Ibãñez C.F., Lendahl U. (2003). Functional Notch signaling is required for BMP4-induced inhibition of myogenic differentiation. Development.

[27] Der Vartanian A., Audfray A., Al Jaam B., Janot M., Legardinier S., Maftah A. (2015). Protein O-fucosyltransferase 1 expression impacts myogenic C2C12 cell commitment via the Notch signaling pathway. Mol. Cell Biol..

[28] Sakai-Takemura F., Nogami K., Elhussieny A., Kawabata K., Maruyama Y., Hashimoto N., Takeda S., Miyagoe-Suzuki Y. (2020). Prostaglandin EP2 receptor downstream of Notch signaling inhibits differentiation of human skeletal muscle progenitors in differentiation conditions. Commun. Biol..

[29] Ma L., Li C., Lian S., Xu B., Yuan J., Lu J., Yang H., Guo J., Ji H. (2021). ActivinA activates Notch1-Shh signaling to regulate proliferation in C2C12 skeletal muscle cells. Mol. Cell Endocrinol..

[30] Fujimaki S., Seko D., Kitajima Y., Yoshioka K., Tsuchiya Y., Masuda S., Ono Y. (2018). Notch1 and Notch2 Coordinately Regulate Stem Cell Function in the Quiescent and Activated States of Muscle Satellite Cells. Stem Cells..

[31] Huot J.R., Marino J.S., Turner M.J., Arthur S.T. (2020). Notch Inhibition via GSI Treatment Elevates Protein Synthesis in C2C12 Myotubes. Biology.

[32] Vergé C., Bouchatal A., Chirat F., Guérardel Y., Maftah A., Petit J.M. (2020). Involvement of ST6Gal I-mediated α2,6 sialylation in myoblast proliferation and differentiation. FEBS Open Bio.

[33] Mourikis P., Sambasivan R., Castel D., Rocheteau P., Bizzarro V., Tajbakhsh S. (2012). A critical requirement for notch signaling in maintenance of the quiescent skeletal muscle stem cell state. Stem Cells.

[34] Fukuda S., Kaneshige A., Kaji T., Noguchi Y.T., Takemoto Y., Zhang L., Tsujikawa K., Kokubo H., Uezumi A., Maehara K. (2019). Sustained expression of HeyL is critical for the proliferation of muscle stem cells in overloaded muscle. Elife.

[35] Al Jaam B., Heu K., Pennarubia F., Segelle A., Magnol L., Germot A., Legardinier S., Blanquet V., Maftah A. (2016). Reduced Notch signalling leads to postnatal skeletal muscle hypertrophy in Pofut1cax/cax mice. Open Biol..

[36] Wen Y., Bi P., Liu W., Asakura A., Keller C., Kuang S. (2012). Constitutive Notch activation upregulates Pax7 and promotes the self-renewal of skeletal muscle satellite cells. Mol. Cell Biol..

[37] Pélisse M., Der Vartanian A., Germot A., Maftah A. (2018). Protein O-Glucosyltransferase 1 Expression Influences Formation of Differentiated Myotubes in C2C12 Cell Line. DNA Cell Biol..

[38] Chen X., Sun Y., Zhang T., Roepstorff P., Yang F. (2021). Comprehensive Analysis of the Proteome and PTMomes of C2C12 Myoblasts Reveals that Sialylation Plays a Role in the Differentiation of Skeletal Muscle Cells. J. Proteome Res..

[39] Skrzypek K., Adamek G., Kot M., Badyra B., Majka M. (2021). Progression and Differentiation of Alveolar Rhabdomyosarcoma Is Regulated by PAX7 Transcription Factor-Significance of Tumor Subclones. Cells.

[40] Esteves de Lima J., Bonnin M.A., Birchmeier C., Duprez D. (2016). Muscle contraction is required to maintain the pool of muscle progenitors via YAP and NOTCH during fetal myogenesis. Elife.

[41] Lahmann I., Bröhl D., Zyrianova T., Isomura A., Czajkowski M.T., Kapoor V., Griger J., Ruffault P.L., Mademtzoglou D., Zammit P.S. (2019). Oscillations of MyoD and Hes1 proteins regulate the maintenance of activated muscle stem cells. Genes Dev..

[42] Saha M., Mitsuhashi S., Jones M.D., Manko K., Reddy H.M., Bruels C.C., Cho K.A., Pacak C.A., Draper I., Kang P.B. (2017). Consequences of MEGF10 deficiency on myoblast function and Notch1 interactions. Hum. Mol. Genet..

[43] Sun H., Li L., Vercherat C., Gulbagci N.T., Acharjee S., Li J., Chung T.K., Thin T.H., Taneja R. (2007). Stra13 regulates satellite cell activation by antagonizing Notch signaling. J. Cell Biol..

[44] Olguín H.C., Pisconti A. (2012). Marking the tempo for myogenesis: Pax7 and the regulation of muscle stem cell fate decisions. J. Cell Mol. Med..

[45] Calhabeu F., Hayashi S., Morgan J.E., Relaix F., Zammit P.S. (2013). Alveolar rhabdomyosarcoma-associated proteins PAX3/FOXO1A and PAX7/FOXO1A suppress the transcriptional activity of MyoD-target genes in muscle stem cells. Oncogene.

[46] AlSudais H., Lala-Tabbert N., Wiper-Bergeron N. (2018). CCAAT/Enhancer Binding Protein β inhibits myogenic differentiation via ID3. Sci. Rep..

[47] Mohamed J.S., Lopez M.A., Cox G.A., Boriek A.M. (2013). Ankyrin repeat domain protein 2 and inhibitor of DNA binding 3 cooperatively inhibit myoblast differentiation by physical interaction. J. Biol. Chem..

[48] Zammit P.S., Relaix F., Nagata Y., Ruiz A.P., Collins C.A., Partridge T.A., Beauchamp J.R. (2006). Pax7 and myogenic progression in skeletal muscle satellite cells. J. Cell Sci..

[49] Nofziger D., Miyamoto A., Lyons K.M., Weinmaster G. (1999). Notch signaling imposes two distinct blocks in the differentiation of C2C12 myoblasts. Development.

[50] Luo D., Renault V.M., Rando T.A. (2005). The regulation of Notch signaling in muscle stem cell activation and postnatal myogenesis. Semin Cell Dev. Biol..

[51] Shawber C., Nofziger D., Hsieh J.J., Lindsell C., Bögler O., Hayward D., Weinmaster G. (1996). Notch signaling inhibits muscle cell differentiation through a CBF1-independent pathway. Development.

[52] Luo Z., Mu L., Zheng Y., Shen W., Li J., Xu L., Zhong B., Liu Y., Zhou Y. (2020). NUMB enhances Notch signaling by repressing ubiquitination of NOTCH1 intracellular domain. J. Mol. Cell Biol..

[53] Girardi F., LeGrand F. (2018). Wnt signaling in skeletal muscle development and regeneration. Prog. Mol. Biol. Transl. Sci..

[54] Amin H., Vachris J., Hamilton A., Steuerwald N., Howden R., Arthur S.T. (2014). GSK3β inhibition and LEF1 upregulation in skeletal muscle following a bout of downhill running. J. Physiol. Sci..

[55] Zhu X.J., Yuan X., Wang M., Fang Y., Liu Y., Zhang X., Yang X., Li Y., Li J., Li F. (2017). A Wnt/Notch/Pax7 signaling network supports tissue integrity in tongue development. J. Biol. Chem..

[56] Gao J., Fan L., Zhao L., Su Y. (2021). The interaction of Notch and Wnt signaling pathways in vertebrate regeneration. Cell Regen..

[57] Kwon C., Cheng P., King I.N., Andersen P., Shenje L., Nigam V., Srivastava D. (2011). Notch post-translationally regulates β-catenin protein in stem and progenitor cells. Nat. Cell Biol..

[58] Pannequin J., Bonnans C., Delaunay N., Ryan J., Bourgaux J.F., Joubert D., Hollande F. (2009). The wnt target jagged-1 mediates the activation of notch signaling by progastrin in human colorectal cancer cells. Cancer Res..

[59] Li B., Jia Z., Wang T., Wang W., Zhang C., Chen P., Ma K., Zhou C. (2012). Interaction of Wnt/β-catenin and notch signaling in the early stage of cardiac differentiation of P19CL6 cells. J. Cell Biochem..

[60] Katoh M. (2006). NUMB is a break of WNT-Notch signaling cycle. Int. J. Mol. Med..

[61] Zhang R., Wang L., He L., Yang B., Yao C., Du P., Xu Q., Cheng W., Hua Z.C. (2014). Fas-Associated Protein with Death Domain Regulates Notch Signaling during Muscle Regeneration. Cells Tissues Organs.

[62] Ge Y., Chen J. (2012). Mammalian target of rapamycin (mTOR) signaling network in skeletal myogenesis. J. Biol. Chem..

[63] Jansen K.M., Pavlath G.K. (2008). Molecular control of mammalian myoblast fusion. Methods Mol. Biol..

[64] Zhang P., Liang X., Shan T., Jiang Q., Deng C., Zheng R., Kuang S. (2015). mTOR is necessary for proper satellite cell activity and skeletal muscle regeneration. Biochem. Biophys. Res. Commun..

[65] Stasiulewicz M., Gray S.D., Mastromina I., Silva J.C., Björklund M., Seymour P.A., Booth D., Thompson C., Green R.J., Hall E.A. (2015). A conserved role for Notch signaling in priming the cellular response to Shh through ciliary localisation of the key Shh transducer Smo. Development.

[66] Liang Y., Han H., Xiong Q., Yang C., Wang L., Ma J., Lin S., Jiang Y. (2021). METTL3-Mediated m6A Methylation Regulates Muscle Stem Cells and Muscle Regeneration by Notch Signaling Pathway. Stem Cells Int..

[67] Gheller B.J., Blum J.E., Fong E.H.H., Malysheva O.V., Cosgrove B.D., Thalacker-Mercer A.E. (2020). A defined N6-methyladenosine (m^6^A) profile conferred by METTL3 regulates muscle stem cell/myoblast state transitions. Cell Death Discov..

[68] Mo C., Zhao R., Vallejo J., Igwe O., Bonewald L., Wetmore L., Brotto M. (2015). Prostaglandin E2 promotes proliferation of skeletal muscle myoblasts via EP4 receptor activation. Cell Cycle.

[69] Ho A.T.V., Palla A.R., Blake M.R., Yucel N.D., Wang Y.X., Magnusson K.E.G., Holbrook C.A., Kraft P.E., Delp S.L., Blau H.M. (2017). Prostaglandin E2 is essential for efficacious skeletal muscle stem-cell function, augmenting regeneration and strength. Proc. Natl. Acad. Sci. USA.

[70] Flamini V., Ghadiali R.S., Antczak P., Rothwell A., Turnbull J.E., Pisconti A. (2018). The Satellite Cell Niche Regulates the Balance between Myoblast Differentiation and Self-Renewal via p53. Stem Cell Rep..

[71] Liu L., Charville G.W., Cheung T.H., Yoo B., Santos P.J., Schroeder M., Rando T.A. (2018). Impaired Notch Signaling Leads to a Decrease in p53 Activity and Mitotic Catastrophe in Aged Muscle Stem Cells. Cell Stem Cell.

[72] Wei D.W., Ma X.Y., Zhang S., Hong J.Y., Gui L.S., Mei C.G., Guo H.F., Wang L., Ning Y., Zan L.S. (2017). Characterization of the promoter region of the bovine SIX1 gene: Roles of MyoD, PAX7, CREB and MyoG. Sci. Rep..

[73] Shan T., Zhang P., Xiong Y., Wang Y., Kuang S. (2016). Lkb1 deletion upregulates Pax7 expression through activating Notch signaling pathway in myoblasts. Int. J. Biochem. Cell Biol..

[74] Kitamoto T., Hanaoka K. (2010). Notch3 null mutation in mice causes muscle hyperplasia by repetitive muscle regeneration. Stem Cells.

[75] Clark B.C., Manini T.M. (2012). What is dynapenia?. Nutrition.

[76] Cohen S., Nathan J.A., Goldberg A.L. (2015). Muscle wasting in disease: Molecular mechanisms and promising therapies. Nat. Rev. Drug Discov..

[77] Li R., Xia J., Zhang X.I., Gathirua-Mwangi W.G., Guo J., Li Y., McKenzie S., Song Y. (2018). Associations of Muscle Mass and Strength with All-Cause Mortality among US Older Adults. Med. Sci. Sports Exerc..

[78] McKenna C.F., Fry C.S. (2017). Altered satellite cell dynamics accompany skeletal muscle atrophy during chronic illness, disuse, and aging. Curr. Opin. Clin. Nutr. Metab. Care.

[79] Egner I.M., Bruusgaard J.C., Gundersen K. (2016). Satellite cell depletion prevents fiber hypertrophy in skeletal muscle. Development.

[80] Narici M.V., Maffulli N. (2010). Sarcopenia: Characteristics, mechanisms and functional significance. Br. Med. Bull..

[81] Domingues-Faria C., Chanet A., Salles J., Berry A., Giraudet C., Patrac V., Denis P., Bouton K., Goncalves-Mendes N., Vasson M.P. (2014). Vitamin D deficiency down-regulates Notch pathway contributing to skeletal muscle atrophy in old wistar rats. Nutr. Metab..

[82] Conboy I.M., Yousef H., Conboy M.J. (2011). Embryonic anti-aging niche. Aging.

[83] Brack A.S., Munoz-Canoves P. (2016). The ins and outs of muscle stem cell aging. Skelet. Muscle.

[84] Sakuma K., Yamaguchi A. (2012). Sarcopenia and age-related endocrine function. Int. J. Endocrinol..

[85] Conboy I.M., Rando T.A. (2012). Heterochronic parabiosis for the study of the effects of aging on stem cells and their niches. Cell Cycle.

[86] Deschenes M.R., Roby M.A., Eason M.K., Harris M.B. (2010). Remodeling of the neuromuscular junction precedes sarcopenia related alterations in myofibers. Exp. Gerontol..

[87] Wang D.X.M., Yao J., Zirek Y., Reijnierse E.M., Maier A.B. (2020). Muscle mass, strength, and physical performance predicting activities of daily living: A meta-analysis. J. Cachexia Sarcopenia Muscle.

[88] McLeod M., Breen L., Hamilton D.L., Philp A. (2016). Live strong and prosper: The importance of skeletal muscle strength for healthy ageing. Biogerontology.

[89] Amarya S.S.K., Sabharwa M.I. (2015). Changes during aging and their association with malnutrition. J. Clin. Gerontol. Geriatr..

[90] Gallagher J.C. (2013). Vitamin D and aging. Endocrinol. Metab. Clin. N. Am..

[91] Latham C.M., Brightwell C.R., Keeble A.R., Munson B.D., Thomas N.T., Zagzoog A.M., Fry C.S., Fry J.L. (2021). Vitamin D Promotes Skeletal Muscle Regeneration and Mitochondrial Health. Front. Physiol..

[92] Srikuea R., Hirunsai M., Charoenphandhu N. (2020). Regulation of vitamin D system in skeletal muscle and resident myogenic stem cell during development, maturation, and ageing. Sci. Rep..

[93] Garcia L.A., King K.K., Ferrini M.G., Norris K.C., Artaza J.N. (2011). 1,25(OH)_2_vitamin D_3_ stimulates myogenic differentiation by inhibiting cell proliferation and modulating the expression of promyogenic growth factors and myostatin in C_2_C_12_ skeletal muscle cells. Endocrinology.

[94] Bass J.J., Nakhuda A., Deane C.S., Brook M.S., Wilkinson D.J., Phillips B.E., Philp A., Tarum J., Kadi F., Andersen D. (2020). Overexpression of the vitamin D receptor (VDR) induces skeletal muscle hypertrophy. Mol. Metab..

[95] Brou C., Logeat F., Gupta N., Bessia C., LeBail O., Doedens J.R., Cumano A., Roux P., Black R.A., Israel A. (2000). A novel proteolytic cleavage involved in Notch signaling: The role of the disintegrin-metalloprotease TACE. Mol. Cell.

[96] Olsson K., Saini A., Stromberg A., Alam S., Lilja M., Rullman E., Gustafsson T. (2016). Evidence for Vitamin D Receptor Expression and Direct Effects of 1alpha, 25(OH)_2_D_3_ in Human Skeletal Muscle Precursor Cells. Endocrinology.

[97] Montenegro K.R., Cruzat V., Carlessi R., Newsholme P. (2019). Mechanisms of vitamin D action in skeletal muscle. Nutr. Res. Rev..

[98] Yousef H., Conboy M.J., Mamiya H., Zeiderman M., Schlesinger C., Schaffer D.V., Conboy I.M. (2014). Mechanisms of action of hESC-secreted proteins that enhance human and mouse myogenesis. Aging.

[99] Carlson M.E., Suetta C., Conboy M.J., Aagaard P., Mackey A., Kjaer M., Conboy I. (2009). Molecular aging and rejuvenation of human muscle stem cells. EMBO Mol. Med..

[100] Carey K., Farnfield M., Tarquinio S., Cameron-Smith D. (2007). Impaired Expression of Notch Signaling Genes in Aged Human Skeletal Muscle. J. Gerontol. Biol. Sci..

[101] Bernet J.D., Doles J.D., Hall J.K., Kelly Tanaka K., Carter T.A., Olwin B.B. (2014). p38 MAPK signaling underlies a cell-autonomous loss of stem cell self-renewal in skeletal muscle of aged mice. Nat. Med..

[102] Chakkalakal J.V., Jones K.M., Basson M.A., Brack A.S. (2012). The aged niche disrupts muscle stem cell quiescence. Nature.

[103] Carlson M., Conboy I.M., Fahy G.M., West M.D., Coles L.S., Harris S.B. (2010). Calibrating Notch/TGF-β Signaling for Youthful, Healthy Tissue Maintenance and Repair. The Future of Aging: Pathways to Human Life Extension.

[104] Pollard T.D., Earnshaw W.C., Lippincott-Schwartz J., Johnson G.T. (2017). Cell Biology.

[105] Sousa-Victor P., Perdiguero E., Munoz-Canoves P. (2014). Geroconversion of aged muscle stem cells under regenerative pressure. Cell Cycle.

[106] Castedo M., Perfettini J.-L., Roumier T., Andreau K., Medema R., Kroemer G. (2004). Cell death by mitotic catastrophe: A molecular definition. Oncogene.

[107] Sinha-Hikim I., Taylor W.E., Gonzalez-Cadavid N.F., Zheng W., Bhasin S. (2004). Androgen receptor in human skeletal muscle and cultured muscle satellite cells: Up-regulation by androgen treatment. J. Clin. Endocrinol. Metab..

[108] Brown D., Hikim A.P., Kovacheva E.L., Sinha-Hikim I. (2009). Mouse model of testosterone-induced muscle fiber hypertrophy: Involvement of p38 mitogen-activated protein kinase-mediated Notch signaling. J. Endocrinol..

[109] Kim J.H., Han G.C., Seo J.Y., Park I., Park W., Jeong H.W., Lee S.H., Bae S.H., Seong J., Yum M.K. (2016). Sex hormones establish a reserve pool of adult muscle stem cells. Nat. Cell Biol..

[110] Seo J.Y., Kang J.S., Kim Y.L., Jo Y.W., Kim J.H., Hann S.H., Park J., Park I., Park H., Yoo K. (2021). Maintenance of type 2 glycolytic myofibers with age by Mib1-Actn3 axis. Nat. Commun..

[111] Zhou X., Wang J.L., Lu J., Song Y., Kwak K.S., Jiao Q., Rosenfeld R., Chen Q., Boone T., Simonet W.S. (2010). Reversal of cancer cachexia and muscle wasting by ActRIIB antagonism leads to prolonged survival. Cell.

[112] Baker Rogers J., Syed K., Minteer J.F. (2021). Cachexia. StatPearls.

[113] Mu X., Agarwal R., March D., Rothenberg A., Voigt C., Tebbets J., Huard J., Weiss K. (2016). Notch Signaling Mediates Skeletal Muscle Atrophy in Cancer Cachexia Caused by Osteosarcoma. Sarcoma.

[114] Lu F., Osei-Hwedieh D., Mandell J.B., Morales-Restrepo A., Hankins M.L., Crasto J.A., Ma R., Dinh V., Watters R.J., Weiss K.R. (2019). Comparison of cachectic and non-cachectic sarcoma patients reveals an important role of Notch signaling in metastasis and myogenesis. Am. J. Cancer Res..

[115] Yoshida T., Galvez S., Tiwari S., Rezk B.M., Semprun-Prieto L., Higashi Y., Sukhanov S., Yablonka-Reuveni Z., Delafontaine P. (2013). Angiotensin II inhibits satellite cell proliferation and prevents skeletal muscle regeneration. J. Biol. Chem..

[116] Acharyya S., Sharma S.M., Cheng A.S., Ladner K.J., He W., Kline W., Wang H., Ostrowski M.C., Huang T.H., Guttridge D.C. (2010). TNF inhibits Notch-1 in skeletal muscle cells by Ezh2 and DNA methylation mediated repression: Implications in duchenne muscular dystrophy. PLoS ONE.

[117] Lin S., Shen H., Jin B., Gu Y., Chen Z., Cao C., Hu C., Keller C., Pear W.S., Wu L. (2013). Brief report: Blockade of Notch signaling in muscle stem cells causes muscular dystrophic phenotype and impaired muscle regeneration. Stem Cells.

[118] Nagata Y., Kiyono T., Okamura K., Goto Y.I., Matsuo M., Ikemoto-Uezumi M., Hashimoto N. (2017). Interleukin-1beta (IL-1β)-induced Notch ligand Jagged1 suppresses mitogenic action of IL-1β on human dystrophic myogenic cells. PLoS ONE.

[119] Gallot Y.S., Straughn A.R., Bohnert K.R., Xiong G., Hindi S.M., Kumar A. (2018). MyD88 is required for satellite cell-mediated myofiber regeneration in dystrophin-deficient mdx mice. Hum. Mol. Genet..

[120] Jeong J., Conboy M.J., Conboy I.M. (2013). Pharmacological inhibition of myostatin/TGF-β receptor/pSmad3 signaling rescues muscle regenerative responses in mouse model of type 1 diabetes. Acta Pharmacol. Sin..

[121] Graham M.L., Janecek J.L., Kittredge J.A., Hering B.J., Schuurman H.J. (2011). The streptozotocin-induced diabetic nude mouse model: Differences between animals from different sources. Comp. Med..

[122] D’Souza D.M., Zhou S., Rebalka I.A., MacDonald B., Moradi J., Krause M.P., Al-Sajee D., Punthakee Z., Tarnopolsky M.A., Hawke T.J. (2016). Decreased Satellite Cell Number and Function in Humans and Mice With Type 1 Diabetes Is the Result of Altered Notch Signaling. Diabetes.

[123] Poddar S., Kesharwani D., Datta M. (2016). Histone deacetylase inhibition regulates miR-449a levels in skeletal muscle cells. Epigenetics.

[124] Poddar S., Kesharwani D., Datta M. (2019). miR-449a regulates insulin signalling by targeting the Notch ligand, Jag1 in skeletal muscle cells. Cell Commun. Signal..

